# Nucleic acid chaperons: a theory of an RNA-assisted protein folding

**DOI:** 10.1186/1742-4682-2-35

**Published:** 2005-09-01

**Authors:** Jan C Biro

**Affiliations:** 1 Homulus Foundation, 88 Howard, #1205; San Francisco, 94 105 CA, USA

## Abstract

**Background:**

Proteins are assumed to contain all the information necessary for unambiguous folding (Anfinsen's principle). However, ab initio structure prediction is often not successful because the amino acid sequence itself is not sufficient to guide between endless folding possibilities. It seems to be a logical to try to find the "missing" information in nucleic acids, in the redundant codon base.

**Results:**

mRNA energy dot plots and protein residue contact maps were found to be rather similar. The structure of mRNA is also conserved if the protein structure is conserved, even if the sequence similarity is low. These observations led me to suppose that some similarity might exist between nucleic acid and protein folding. I found that amino acid pairs, which are co-located in the protein structure, are preferentially coded by complementary codons. This codon complementarity is not perfect; it is suboptimal where the 1st and 3rd codon residues are complementary to each other in reverse orientation, while the 2nd codon letters may be, but are not necessarily, complementary.

**Conclusion:**

Partial complementary coding of co-locating amino acids in protein structures suggests that mRNA assists in protein folding and functions not only as a template but even as a chaperon during translation. This function explains the role of wobble bases and answers the mystery of why we have a redundant codon base.

## Introduction

The protein folding problem has been one of the grand challenges in computational molecular biology. The problem is to predict the native three-dimensional structure of a protein from its amino acid sequence. It is widely believed that the amino acid sequence contains all the necessary information for the correct three-dimensional structure, since protein folding is apparently thermodynamically determined; i.e., given a proper environment, a protein will fold spontaneously to the correct conformation. This is called Anfinsen's thermodynamic principle [1].

The thermodynamic principle has been confirmed many times on many different kinds of proteins in vitro. Critics says that the in vivo chemical conditions are different from those in vitro, correct protein folding is determined by interactions with other molecules (chaperons, hormones, substrate, etc.) and is much more complex than renaturation of denatured poly amino acids. The fact that many naturally-occurring proteins fold reliably and quickly to their native states, despite the astronomical number of possible configurations, has come to be known as Levinthal's paradox [2].

Anfinsen's principle was formulated in the 1960s using purely chemical experiments and a lot of intuition. Today, many sequences and structures are available to establish a logical and understandable link between sequence, structure and function. But it is still not possible to predict the structure (or a range of possible structures) correctly from the sequence alone, ab initio and in silico [3].

There are two potential, external sources of additional and specific protein folding information: (a) the chaperons (other proteins that assist in the folding of proteins and nucleic acids [4]; and (b) the protein-coding nucleic acid sequences themselves (which are templates for protein syntheses, but are not defined as chaperons). Protein chaperons are not necessarily similar to their clients; they can be complementary templates, too, as it is well known from nucleic acid interactions. However, chaperons necessarily contain spatial information (in some form) that guides another protein to fold correctly. Chaperoning requires subtle interactions with the immaturely folded intermediate so that its structure is loosened and it is then released for successive rounds of folding attempts. (Some aspects of this situation might be compared to enzyme-substrate interactions and kinetics.)

The possibility that the nucleotide sequence itself could modulate translation and hence affect co-translational folding and assembly of proteins has been investigated in a number of studies [5-7]. Studies on the relationships between synonymous codon usage and protein secondary structural units are especially popular [8-10]. The genetic code is redundant (61 codons encode 20 amino acids) and as many as six synonymous codons can encode the same amino acid (Arg, Leu, Ser). The "wobble" base has no effect on the meaning of most codons, but codon usage (wobble usage) is nevertheless not randomly defined [11,12] and there are well-known, stable species-specific differences in codon usage. It seems to be reasonable to search for the meaning (biological purpose) of the wobble bases in association with protein folding.

## Materials and methods

We have developed a tool, SeqX [13], which is specially designed to provide 2D projections of protein structures (residue contact maps) and analyze residue co-locations statistically in these structures. We have collected residue co-location statistics (residue contact statistics) from 80 different structures from the Protein Data Bank (PDB) [14]. This non-redundant SeqX data set listed ~35,000 amino acid co-locations (i.e. residues located within a 6 Å radius of the alpha carbon atoms; neighbor residues on the same strand were excluded).

The mfold tool was used to obtain RNA structure data [15] and the energy dot-plots provided by this program were used to estimate the site and size of the most probable RNA folding.

Student's *t*-tests were used for statistical evaluation.

## Results and Discussion

The very first idea of protein folding on a nucleic acid template was the model of direct protein synthesis on the surface of dsDNA. It was suggested by George Gamow [16,17] before the discovery of the genetic code and mRNA. Gamow noticed that the distances between base pairs in DNA and the distances between amino acids in proteins are the same (~4 Å), and he suggested that complementary base pairs formed 20 different "cavities" on the surface of the DNA (one for each amino acid) where the amino acid residues aligned and became ligated. The correct translation turned out to be mRNA-mediated and stereochemical fitting between DNA and protein residues was rejected [18].

However, this question arose again in a different form. Specific DNA-protein interactions do exist (such as those between DNA and transcription factors or between restriction enzymes and recognition sequences), and it is difficult to explain the extreme specificity of these interactions without assuming that there is a "small scale", "residue level" interaction between nucleic acids and proteins. I found Woese's idea [19] of stereochemical fitting very attractive, i.e. affinity between codons and coded amino acids, in contrast to Crick's statement of a "frozen accident" [18]. I succeeded in constructing *A Common Periodic Table of Codons and Amino Acids *[20] and in showing a large number of codon-amino acid co-locations in restriction enzyme-recognition sequence structures [21]. Consequently, I support the view that the unit of specific nucleic acid-protein interactions is the codon and its amino acid.

Nucleic acids are structure-forming molecules. Perfect complementarity between Watson and Crick (WC) base pairs forms the perfect helical structure, dsDNA. However, partial or suboptimal WC complementarity in and between strands provides a large number of DNA/RNA structure variations. The structural variation of a given RNA might be large; some structures are energetically more favored, some are less. The importance of one RNA secondary structure over another is usually not a subject of debate, because RNA structure often has no known physiological significance (there are exceptions, e.g. tRNAs).

Proteins are also structure-forming molecules. However, in contrast to nucleic acids, there is no known specific amino acid complementarity, and the known physicochemical rules (charge, hydrophobe, size compatibility) are often insufficient to define only one obvious protein folding and structure (Biro, 2005, unpublished). The limitation of Anfinsen's theorem [1] is described by the Levinthal paradox [2], which is confirmed by the often frustrating outcome of ab initio protein prediction. However, we know that there is very little biological tolerance for variation in protein structure; usually only one main functioning structure is assigned to a protein sequence (and sometimes a few allosteric variants). The exact structure of a protein is critical, as is evident from our knowledge of prions. However, the primary sequence is usually insufficient to establish this exact structure and chaperons are required. The problem is not that there is a large choice of different protein folding pathways with different end-points, only one of which is physiologically normal. Rather, the problem is the risk of deviation from *the *(physiological) folding pathway to form any one of a number of misfolded molecules. Chaperones are needed because the sequence is insufficient to define the most effective folding pathway leading to the thermodynamically most stable structure.

**Figure 1:**
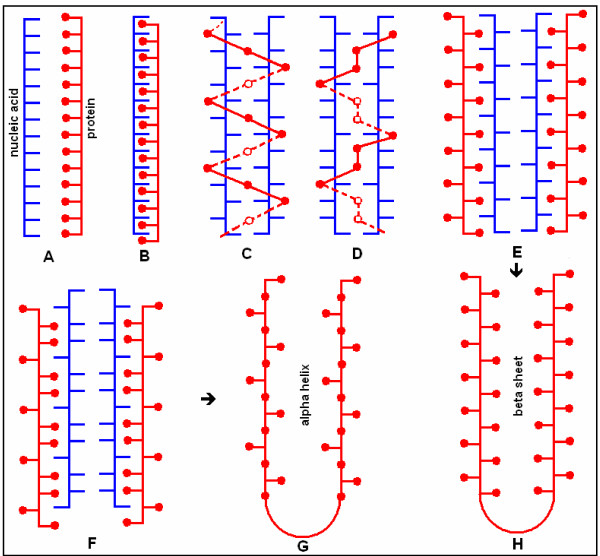
Nucleo-protein structures and protein foldings. The distance between bases in nucleic acids (horizontal blue lines) and amino acids in proteins (red dots) is almost the same (A). This suggests the possibility of residue-level interactions between these molecules (B). Partial, sub-optimal complementarity between DNA strands ("honeycomb structure") and fitting of amino acids into DNA cavities was suggested by Gamow [16, 17] (C, D). A further development of this model is that partial complementarity between mRNA subsequences (E, F) determines the orientation of amino acid residues in ribonucleoprotein complexes and consequently the RNA loops serve as templates (RNA chaperons) to main secondary protein structures such as alpha helices (G) and beta-sheets (H). Codon boundaries are not indicated in these models. The Figure illustrates the historical development of the concept of direct and specific nucleic acid – protein interactions and its possible consequences for protein folding.

Chaperons are defined as proteins of which the function is to assist the folding of other proteins. However, the most obvious chaperons for me are nucleic acids; specifically, those coding the protein in question (Fig. 1). Immediate RNA-assisted protein folding prevents any protein misfolding at the site of protein synthesis itself. The insufficiency of folding information in protein sequences is more than compensated by the excess of information (codon base redundancy) in nucleic acids.

**Figure 2:**
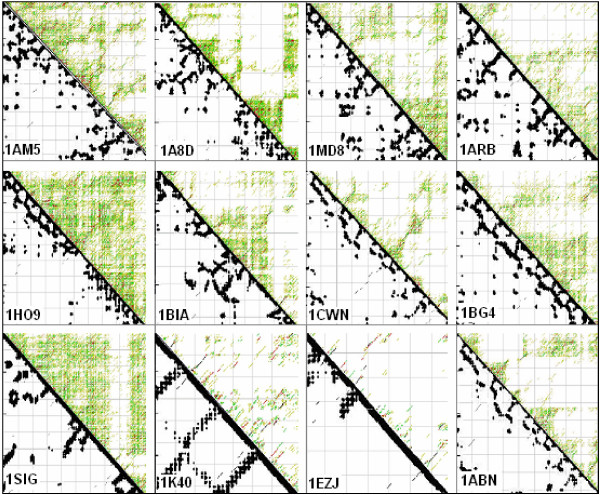
Comparison of 12 randomly selected protein and corresponding mRNA structures. Residue contact maps (RCM) were obtained from the PBD files of the protein structures using the SeqX tool (left triangles). Energy dot plots (EDP) for the coding sequences were obtained using the mfold tool (right triangles). The two maps were aligned along a common left diagonal axis to facilitate visual comparison between the different possible representations. The black dots in the RCMs indicate amino acids that are within 6 Å of each other in the protein structure. The colored (grass-like) areas in the EDPs indicate the energetically mostly likely RNA interactions (color code in increasing order: yellow, green red, black). The full names and the lengths of the proteins (number of amino acid residues): 1AM5: PEPSIN (324), 1A8D: TETANUS NEUROTOXIN (451), 1MD8: SERIN PROTEASE (329), 1ARB: ACHROMOBACTER PROTEASE I (268), 1HO9: A ALPHA-2A ADRENERGIC RECEPTOR (32), 1BIA: BIRA BIFUNCTIONAL PROTEIN (376), 1CWN: ALDEHYDE REDUCTASE (324), 1BG4: ENDO-1,4-BETA-XYLANASE (302), 1SIG: RNA POLYMERASE PRIMARY SIGMA FACTOR (339) bases, 1K40: ADHESION KINASE (126), 1EZJ: NUCLEOCAPSID PHOSPHOPROTEIN (140), 1ABN: ALDOSE REDUCTASE (315). The coordinates indicate the number of amino acid and the corresponding nucleic acid residues.

I compared the structures of mRNAs with those of the translated proteins to test the assumption that protein folding information is present in mRNA. The energy dot-plots provided by mfold and the 2D protein structures provided by SeqX indeed suggest similarity in most of the randomly selected structures (Fig. 2)

**Figure 3:**
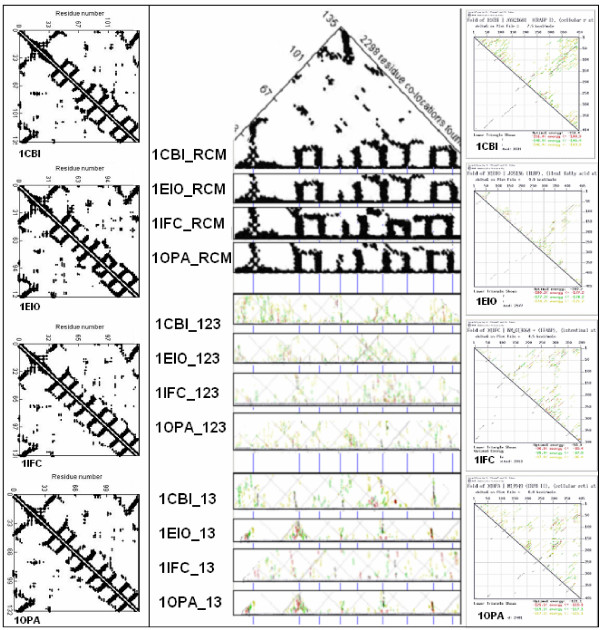
Comparison of the protein and mRNA secondary structures. Residue contact maps (RCM) were obtained from the PBD files of four protein structures (1CBI, 1EIO, 1IFC, 1OPA) using the SeqX tool (left column). Energy dot plots (EDP) for the coding sequences were obtained using the mfold tool (right column). The left diagonal portions of these two maps are compared in the central part of the figure. Blue horizontal lines in the background correspond to the main amino acid co-location sites in the RCM. Intact RNA (123) as well as subsequences containing only the 1st and 3rd codon letters (13) are compared. The black dots in the RCMs indicate amino acids that are within 6 Å of each other in the protein structure. The colored (grass-like) areas in the EDPs indicate the energetically most likely RNA interactions (color code in increasing order: yellow, green red, black). The full names and the lengths of the proteins (number of amino acid residues): 1CBI: CELLULAR RETINOIC ACID BINDING PROTEIN I (136), 1EIO: ILEAL LIPID BINDING PROTEIN (127), 1IFC: INTESTINAL FATTY ACID BINDING PROTEIN (132), 1OPA: CELLULAR RETINOL BINDING PROTEIN II (135).

Another similar, but still not quantitative, comparison of protein and coding structures was performed on four proteins that are known to have very similar 3D structures although their primary structures (sequences) are less than 30% similar, and on the sequences of their mRNAs. These four proteins exemplify the fact that protein tertiary structure is much more conserved than the amino acid sequence. I asked whether this is also true for RNA structures and sequences. I found that there are signs of conservation even of RNA secondary structure (as indicated by the energy dot plots) and there are similarities between the protein and nucleic acid structures (Figure 3).

These structural comparisons are suggestive, but not quantitative, and more convincing statistical evaluation is necessary to evaluate the significance of the suggested similarity between nucleic acid and corresponding protein structures. (Quantitative comparisons of 2D protein representations and RNA energy dot plots are possible and are in progress in our laboratory). Similarities between two macromolecules (RNA-RNA, protein-protein, RNA-protein), or even between two macromolecular families, does not automatically mean that they are functionally related to each other (or that one is a chaperon), but it is a widely accepted sign of a biologically significant relationship.

The molecular basis of mRNA structure formation is the known WC base pair complementarity. Therefore I asked whether it is possible to find some kind of complementarity between the codons of co-locating (specifically interacting) amino acids.

**Figure 4:**
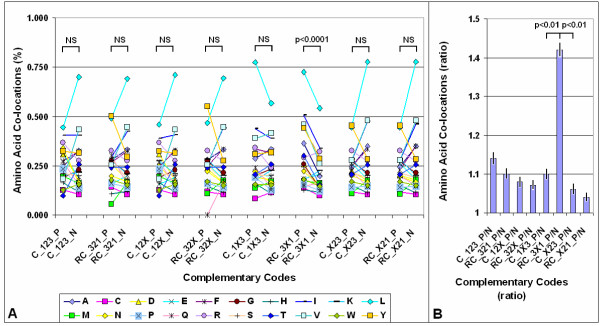
Complementary codes vs. amino acid co-locations. (A) The propensity of the 400 possible amino acid pairs was monitored in 80 different protein structures with the SeqX tool. The tool detected co-locations when two amino acids were closer than 6 Å to each other (neighbors on the same strand were excluded). The total number of co-locations was 34,630. Eight different complementary codes were constructed for the codons (two optimal and six suboptimal). In the two optimal codes all three codon residues (123) were complementary (C) or reverse-complementary (RC) to each other. In the suboptimal codes only two of three codon residues were C or RC to each other (12, 13, 23), while the third was not necessarily complementary (X). (For example, complementary code RC_1X3 means that the first and third codon letters are always complementary, but not the second, and the possible codons are read in reverse orientation). The 400 co-locations were divided into 20 subgroups corresponding to 20 amino acids (one of the co-locating pairs), each group containing the 20 amino acids (corresponding to the other amino acid in the co-locating pair). If the codons of the amino acid pairs followed the predefined complementary code, the co-location was regarded as positive (P); if not, the co-location was regarded as negative (N). Each symbol represents the mean frequency of P or N co-locations corresponding to the indicated amino acid. Paired Student's *t*-test, *n *= 20 (see Fig. 2 for explanation). (B) The ratio of positive (P) and negative (N) co-locations was calculated on data from (A). Each bar represents the mean ± SEM, *n *= 20.

Searching for some pattern in the codons of co-locating amino acids, the frequency of the eight possible patterns in the 64 nucleic acid triplets was analyzed. The codons were either complementary to each other in all three (-123-) or in at least two codon base positions (-12X-, -1X3-, -X23-). In these latter cases the codon complementarity was partial, because complementarity was not required for one position (X). The complementary codons were translated in the same (5' > 3' and 3' > 5', only complementary, C) or the reversed and complementary (5' > 3' and 5' > 3', RC) directions. One (and only one) codon complementary pattern of the eight possible turned out to be significantly overrepresented among the codons of co-locating amino acids: D-1X3/RC-3X1. The other 7 possible codon patterns served as negative controls. This pattern means that the 1st and 3rd codon residues are complementary in reverse orientation, but the 2nd residue may be but is not necessarily complementary (X) (Fig. 4). The possible amino acid pairs determined by the D-1X3/RC-3X1 formula are indicated in Table I.

**Table I:** Amino Acids Coded by Partially Complementary Codons

**D_1X3/RC_3X1**			1st	G	T	G	G	T	G	C	A	A	CT	A	A	C	C	AC	AT	A	G	T	T
			
			2nd	C	G	A	A	T	G	A	T	A	T	T	A	C	A	G	CG	C	T	G	A
			
			3rd	X	CT	CT	AG	CT	X	CT	ACT	AG	XAG	G	CT	X	AG	AGX	CTX	X	X	G	CT
1st	2nd	3rd	AA	**A**	**C**	**D**	**E**	**F**	**G**	**H**	**I**	**K**	**L**	**M**	**N**	**P**	**Q**	**R**	**S**	**T**	**V**	**W**	**Y**
G	C	X	**A**	+	+	+		+	+	+	+		+		+	+		+	+	+	+		+
T	G	CT	**C**	+			+		+		+	+						+		+	+		
G	A	CT	**D**	+		+			+		+				+				+	+	+		
G	A	AG	**E**		+			+		+			+			+		+	+				+
T	T	CT	**F**	+			+		+		+	+						+		+	+		
G	G	X	**G**	+	+	+		+	+	+	+		+		+	+		+	+	+	+		+
C	A	CT	**H**	+			+		+			+		+				+		+	+		
A	T	ACT	**I**	+	+	+		+	+		+				+				+	+	+		+
A	A	AG	**K**		+			+		+			+			+		+	+				+
CT	T	XAG	**L**	+			+		+			+	+	+		+	+	+	+	+	+	+	
A	T	G	**M**							+			+			+		+					
A	A	CT	**N**	+		+			+		+				+				+	+	+		
C	C	X	**P**	+			+		+			+	+	+		+	+	+	+	+	+	+	
C	A	AG	**Q**										+			+	+	+	+			+	
AC	G	AGX	**R**	+	+		+	+	+	+		+	+	+		+	+	+	+	+	+	+	+
AT	CG	CTX	**S**	+		+	+		+		+	+	+		+	+	+	+	+	+	+		
A	C	X	**T**	+	+	+		+	+	+	+		+		+	+		+	+	+	+		+
G	T	X	**V**	+	+	+		+	+	+	+		+		+	+		+	+	+	+		+
T	G	G	**W**										+			+	+	+					
T	A	CT	**Y**	+			+		+		+	+						+		+	+		

RC_3X1 code: 1st and 3rd codon letters are complementary in reverse order, indicated by complementary colors (red, blue); X: any residue; AA: amino acids, one-letter code, +: AAs coded by the D_1x3/RC_3X1 complementary codons

This partial, suboptimal complementarity again suggests that mRNA folding may assist protein folding, but does not necessarily prove it. An alternative explanation is that it is only a sign of the biochemical origin of specifically interacting amino acid pairs (they are encoded in partially complementary codons) but does not mean that complementary structures in amino acids will form interacting protein strands.

**Figure 5:**
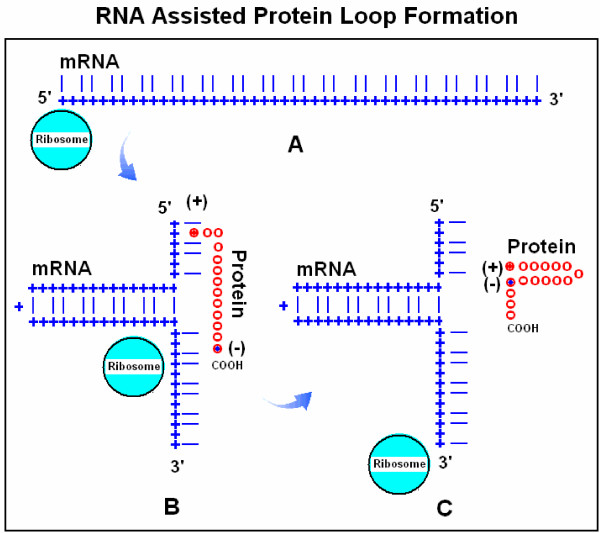
RNA assisted protein loop formation. Translation begins with the attachment of the 5' end of a mRNA to the ribosome (A). Ribonucleotides are indicated by blue + and the 1^st ^and 3^rd ^bases in the codons by blue lines, while the 2^nd ^base positions are left empty. A positively charged amino acid [(+) and red dots], for example arginine, remains attached to its codon. The mRNA forms a loop because the 1^st ^and 3^rd ^bases are locally complementary to each other in reverse orientation (B). The growing protein is indicated by red circles (o). When translation proceeds to an amino acid with especially high affinity to the mRNA-attached arginine, for example a negatively charged Glu or Asp [(-) and blue dot], the charge attraction removes the Arg from its mRNA binding site and the entire protein is released from the mRNA and completes a protein loop (C). The protein continues to grow toward the direction of its carboxy terminal (COOH).

**Figure 6:**
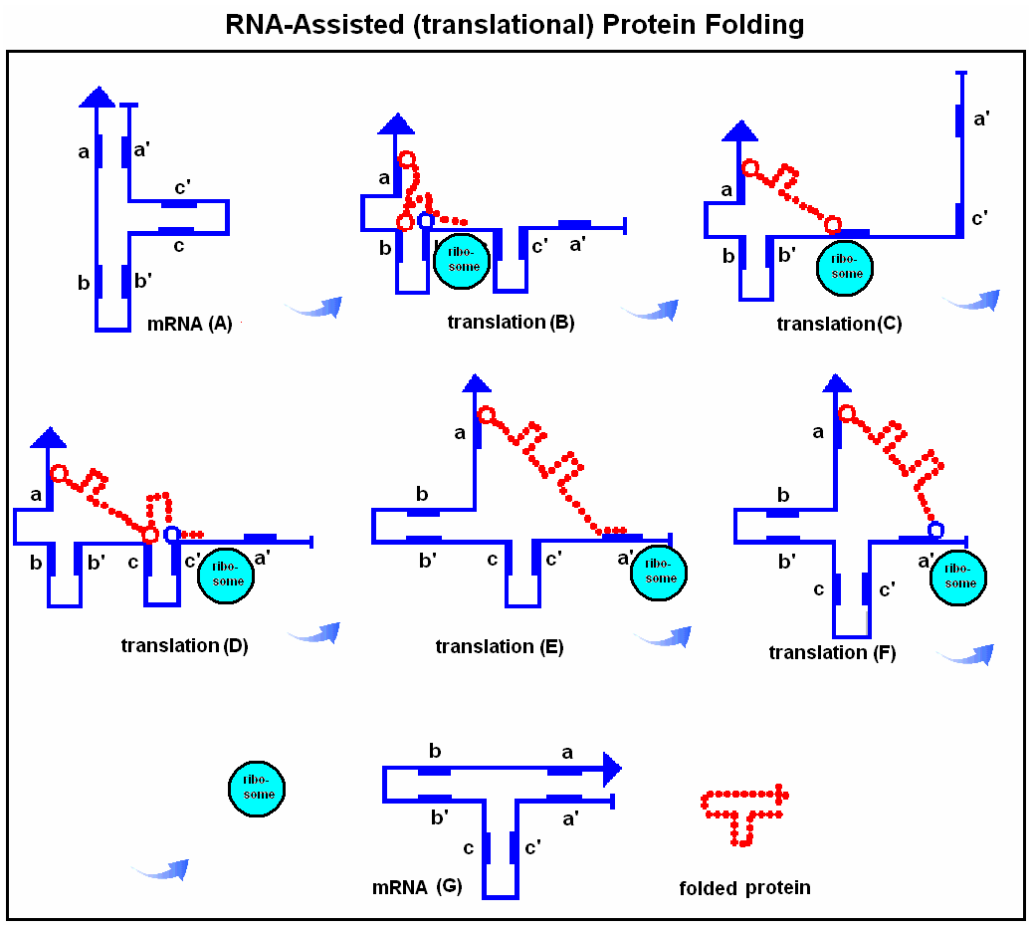
RNA-assisted (translational) protein folding. There are three reverse and complementary regions in a mRNA (blue line, A): a-a', b-b', c-c', which fold the mRNA into a T-like shape. During the translation process the mRNA unfolds on the surface of the ribosome, but subsequently refolds, accompanied by its translated and lengthening peptide (red dotted line, B-F). The result of translation is a temporary ribonucleotide complex, which dissociates into two T-shape-like structures: the original mRNA and the properly folded protein product (G). The red circles indicate the specific, temporary attachment points between the RNA and protein (for example a basic amino acid) while the blue circles indicate amino acids with exceptionally high affinity for the attachment points (for example acidic amino acids); these capture the amino acids at the attachment point and dissociate the ribonucleoprotein complex. Transfer-RNAs are of course important participants in translation, but they are not included in this scenario.

The historical concept of specific nucleic acid – protein interactions and the subsequent possibility of RNA-assisted protein folding was illustrated in figure 1. I wish to suggest a further development of these ideas. The distance between codons is about three times larger than the distance between amino acids and therefore complete 1 by 1 RNA-protein alignment is not possible. Furthermore, a long continuous alignment would create problems in dissociating the nucleoprotein complexes. Therefore I suggest that only some basic (positively charged) amino acids remain attached to their codons (or become re-attached after removal of tRNA). If this attachment point is followed by a loop in the mRNA, a corresponding loop will be formed in the nascent protein (Figure 5). The interaction between the positively charged amino acid and the negatively charged codon will be successively weakened by the growing protein loop and finally interrupted, for example, by the translation of a negatively charged amino acid. It is known that interactions between nucleic acids and proteins often involve only a few amino acids and that these "patchy" interaction sites often contain an arginine [21]. Complex protein structures might be folded in this way (Figure 6).

The observed partial complementary coding of co-locating amino acids (the D_1X3/RC_3X1 formula) raises a series of interesting questions. The 20 amino acid – triplet codon model, obviously entails the need for a third codon base (two nucleotides are simply not enough). However, based on the assumption of RNA chaperons, two proteins with identical primary structures (for example human and chimpanzee Hb) may fold differently if there are differences in the redundant codon base positions. Similarly, a number of SNPs (Single Nucleotide Polymorphisms) that do not change the coded amino acids may result in protein structure variations.

The medical genetics literature (for example OMIM) is full of annotations concerning wobble base mutations and it is usually inferred that these "translationally silent" mutations are unlikely to cause disease. A famous exception is prion diseases (mad cow disease, Creutzfeldt-Jakob disease [22]). This large group of diseases is characterized by the presences of an abnormally folded protein (PrP^sc^) instead of the normally folded one (PrP^C^). The physiological and abnormal proteins have the same primary structures; only the secondary structures are different. In most cases the disease is acquired by infection, but there are many inherited forms. At least 42 known point mutations, 24 causative and 18 translationally silent, are described in the literature [23]. The wobble base mutations demand serious attention, especially since it is known that selection pressure exists for the wobble bases in some codon positions [24].

The RNA chaperon theory does not mean that every wobble-base point-mutation (or SNP) influences secondary structure. Usually, many codons and amino acids are involved in the formation of a simple secondary structure element (helix, sheet, turn) and probably most mutations have no structural consequences. Also, many mutations are accompanied by a second, compensatory mutation that corrects the structural consequences of the first. In evolution, sequence changes more rapidly than structure; however, many sequence changes are compensatory and preserve local physicochemical characteristics. For example, if an amino acid side chain is particularly bulky with respect to the average at a given position in a given sequence, this might have been compensated in evolution by a particularly small side chain in a neighbouring position, preserving the general structural motif [25].

An additional question raised by the RNA-chapeon hypotheses concerns the GC versus AT contents of various genomes, which range from 78 / 22 to 22 / 76. This causes marked differences, especially in the compositions of the third codon nucleotides. It is reasonable to suppose that redundant codon bases are susceptible to much more variation if there is no amino acid replacement, and that if such changes affect protein folding, this would have restrained such nucleotide replacements significantly. However this is not necessarily true. The partial complementary coding of co-locating amino acids (the D_1X3/RC_3X1 rule) suggests that the number of possible amino acid co-locations is less than 200 (20 × 20/2), and the possible co-locations involve pairings of physicochemically compatible amino acids (Biro, 2005, unpublished). Many non-silent mutations in one codon are coupled to a second (silent or non-silent) mutation in a second codon. This coupled and coordinated model of mutations actually permits a very large number of variations in the primary nucleic acid and protein sequences with no consequences for nucleic acid or protein secondary structures. And as indicated above, 3D structures are generally much more conserved than sequences.

Complementary coding of co-locating amino acids, and the consequent possibility of nucleic acid assisted protein folding (nucleic acid chaperon), might give new insights into the dilemma of why we have a redundant codon base and might explain the role of the wobble base in the codon. Experimental, in vitro support is necessary to confirm this in silico suggestion of nucleic acid chaperons.

## Declaration of competing interests

The author(s) declare that they have no competing interests.
